# Rifampin modulation of xeno‐ and endobiotic conjugating enzyme mRNA expression and associated microRNAs in human hepatocytes

**DOI:** 10.1002/prp2.386

**Published:** 2018-03-26

**Authors:** Brandon T. Gufford, Jason D. Robarge, Michael T. Eadon, Hongyu Gao, Hai Lin, Yunlong Liu, Zeruesenay Desta, Todd C. Skaar

**Affiliations:** ^1^ Department of Medicine Division of Clinical Pharmacology Indiana University School of Medicine Indianapolis IN; ^2^ Department of Medical and Molecular Genetics Indiana University School of Medicine Indianapolis IN

**Keywords:** drug metabolizing enzyme induction, miRNA modulation of mRNA, PBPK modeling, phase 2 enzyme induction, rifampin miRNA induction, rifampin mRNA repression

## Abstract

Rifampin is a pleiotropic inducer of multiple drug metabolizing enzymes and transporters. This work utilized a global approach to evaluate rifampin effects on conjugating enzyme gene expression with relevance to human xeno‐ and endo‐biotic metabolism. Primary human hepatocytes from 7 subjects were treated with rifampin (10 μmol/L, 24 hours). Standard methods for RNA‐seq library construction, EZBead preparation, and NextGen sequencing were used to measure UDP‐glucuronosyl transferase UGT, sulfonyltransferase SULT, N acetyltransferase NAT, and glutathione‐S‐transferase GST mRNA expression compared to vehicle control (0.01% MeOH). Rifampin‐induced (>1.25‐fold) mRNA expression of 13 clinically important phase II drug metabolizing genes and repressed (>1.25‐fold) the expression of 3 genes (*P *<* *.05). Rifampin‐induced miRNA expression changes correlated with mRNA changes and miRNAs were identified that may modulate conjugating enzyme expression. NAT2 gene expression was most strongly repressed (1.3‐fold) by rifampin while UGT1A4 and UGT1A1 genes were most strongly induced (7.9‐ and 4.8‐fold, respectively). Physiologically based pharmacokinetic modeling (PBPK) was used to simulate the clinical consequences of rifampin induction of CYP3A4‐ and UGT1A4‐mediated midazolam metabolism. Simulations evaluating isolated UGT1A4 induction predicted increased midazolam N‐glucuronide exposure (~4‐fold) with minimal reductions in parent midazolam exposure (~10%). Simulations accounting for simultaneous induction of both CYP3A4 and UGT1A4 predicted a ~10‐fold decrease in parent midazolam exposure with only a ~2‐fold decrease in midazolam N‐glucuronide metabolite exposure. These data reveal differential effects of rifampin on the human conjugating enzyme transcriptome and potential associations with miRNAs that form the basis for future mechanistic studies to elucidate the interplay of conjugating enzyme regulatory elements.

**Abbreviations**
CYP: cytochrome P450
DMSO: dimethyl sulfoxide
GEO: Gene Expression Omnibus
GST: glutathione‐S‐transferase
miRNA: microRNA
NAPQI: N‐acetyl‐p‐benzoquinone imine
NAT: N acetyltransferase
PBPK: physiologically based pharmacokinetic
PXR: pregnane X receptor
SULT: sulfonyltransferase
TPMT: thiopurine S‐methyltransferase
UGT: UDP‐glucuronosyl transferase

## 1 INTRODUCTION

Rifampin induction of cytochrome P450 is an extensively studied drug–drug interaction mechanism resulting in a substantial list of clinically important interactions that can lead to reduced drug efficacy or increased toxicity.^1^, ^2^ In contrast, relatively less is known about rifampin induction of human conjugating enzymes including uridine diphosphate glucuronosyltransferases (UGTs), sulfotransferases (SULTs), N‐acetyltransferases (NATs), thiopurine S‐methyltransferase (TPMT) and glutathione S‐transferases (GSTs).^3^ Rifampin is widely recognized as a pleiotropic but specific inducer of drug metabolizing enzymes and transporters with effects mediated mainly through activation of pregnane X receptor (PXR).^4^ The genes regulated by PXR include those encoding for human conjugating enzyme families (UGTs, SULTs, NATs, and GSTs). Previous studies demonstrated rifampin induction of miRNAs and association with repression of P450 genes, suggesting the possibility of additional epigenetic mechanisms underlying rifampin drug–drug interactions.^5^, ^6^ Epigenetic modulation of conjugating enzymes by miRNAs has also been demonstrated.^7^, ^8^, ^9^, ^10^ MiRNAs generally are thought to negatively regulate gene expression and reduce downstream protein translation via imperfect complementary binding with the 3′‐untranslated region. However, relatively little is known about the combined effects of rifampin‐induced changes in hepatic miRNA expression on the downstream expression of conjugating enzymes.

The UGT superfamily of conjugating enzymes contains 5 subfamilies (UGT1, UGT2A, UGT2B, UGT3, and UGT8). Three of these subfamilies (UGT1, UGT2A, and UGT2B) prominently contribute to the metabolism of drugs, dietary substances, toxicants, and endogenous substrates with broad and overlapping substrate specificities. These 3 subfamilies are encoded by 10 genes to generate 19 isoforms in humans.^11^ The UGT1A family shares a single chromosomal locus (band 2q37) with the 9 different functional isoforms being generated via splicing of shared exons 2‐5 to an isoform‐specific exon 1. Similarly, the UGT2A subfamily members share exons 2‐6 with an isoform‐specific exon 1. Conversely, the UGT2B family is composed of 7 functional enzymes encoded by individual genes. Each UGT possesses a unique 5′‐upstream promoter region that controls its transcription as well as more distant enhancer regions containing transcription factor‐binding sites that further control constitutive and inducible UGT expression. A wide variety of tissue‐specific and ligand‐activated transcription factors modulate the induction of UGT genes including PXR.^12^ In addition, epigenetic UGT regulation by miRNAs has recently been identified as another factor that modulates UGT expression and response to environmental exposures.^7^, ^8^, ^9^, ^10^, ^13^, ^14^ Taken together, evaluating the influence of rifampin on UGT mRNA expression and association with miRNA changes may help to unravel the complex regulatory network governing UGT expression and activity.

The cytosolic SULT family of enzymes contribute to the metabolism of several exogenous and endogenous substrates, including the clinically used drugs acetaminophen, minoxidil, and ethinyl estradiol. The SULT family is comprised of 13 members within 3 families (SULT1, SULT2, and SULT4). SULT activity varies widely among individuals due in part to genetic polymorphisms and susceptibility to induction via nuclear receptor activation.^15^, ^16^, ^17^ For women taking ethinyl estradiol, rifampin induction of SULTs may cause therapeutic failure of the oral contraceptive drug.^18^ Despite the clinical importance of SULT‐mediated xenobiotic metabolism, data describing mechanisms regulating SULT induction are rather sparse.

NATs, another family of conjugating enzymes, contribute to human xenobiotic and endogenous substrate metabolism. Two NATs, NAT1 and NAT2, are thought to be of primary importance to drug metabolism. Polymorphisms exist in both NAT1 and NAT2 genes with well‐established functional consequences in phenotypic slow acetylators. For example, slow acetylators are more susceptible to drug‐induced toxicities from hydralazine and isoniazid. Isoniazid and rifampin are also commonly coadministered for the treatment of latent tuberculosis, raising the potential for drug–drug interactions. Slow acetylators are also more prone to developing certain cancers.^19^ As a result, NAT modulation via small molecules and miRNAs has become a target of drug and biomarker development.^20^, ^21^ Considered together, understanding the rifampin‐induced changes in NAT expression and associated miRNAs may be of therapeutic and diagnostic value.

TPMT is the primary enzyme responsible for human metabolism of thiopurine drugs including azathioprine, thioguanine, and 6‐mercaptopurine. Genetic polymorphisms in TMPT can result in reduced enzyme activity leading to increased drug concentration and toxicities in certain patients. As a result, pharmacogenetics screening for TPMT deficiency is recommended prior to initiating thiopurine drug therapy. A previous report demonstrated no change in TPMT mRNA expression in human hepatocytes treated with rifampin^3^ but the potential influence of miRNAs has not been previously explored.

Human GSTs are a family of cytosolic enzymes that catalyze the transfer of the sulfhydryl group of glutathione to a large variety of electrophiles, including drug molecules such as busulfan and ethacrynic acid and reactive CYP450 metabolites such as N‐acetyl‐p‐benzoquinone imine (NAPQI). GST induction by drug molecules and dietary flavonoids has been previously reported^22^, ^23^ but the potential relationship with miRNA expression changes has not been evaluated.

The first aim of this report was to describe the effects of rifampin treatment on the regulation of hepatic conjugating enzyme mRNA expression and the relationships with regulation of miRNA expression in primary human hepatocytes. The second aim was to further assess the impact of rifampin modulation of UGT mRNA expression in human renal proximal tubule cells to evaluate the potential for tissue‐specific changes in enzyme regulation. Finally, based upon the in vitro and in silico study results, rifampin induction of UGT1A4‐mediated metabolism was selected for further evaluation via physiologically based pharmacokinetic (PBPK) modeling and simulation. The overarching goal of this work was to globally evaluate rifampin's effects on conjugating enzyme gene expression with relevance to human xeno‐ and endobiotic metabolism.

## 2 MATERIALS AND METHODS

### 2.1 Primary human hepatocytes and drug treatments

This study evaluated mRNA and miRNA expression data collected in a previously published human hepatocyte experiment.^5^, ^6^ In brief, freshly isolated human hepatocytes from 7 different donors were obtained from CellzDirect (Durham, NC) and were plated on 12‐well collagen‐coated plates cultured in Williams’ E medium without phenol red containing Primary Hepatocyte Maintenance Supplements (Life Technologies Corporation, Carlsbad, CA). Cultures from each donor were considered biological replicates (n = 7). All studies were performed within 72 and 120 hours following the time of hepatocyte isolation. Hepatocytes were treated with rifampin (10 μmol/L) or corresponding vehicle control (0.1% methanol) for 24 hours. The commercially obtained human hepatocytes were deidentified and specific demographic and/or clinical information were not available from the supplier.

### 2.2 MicroRNA expression profiling and bioinformatics analysis

Total RNA, including small RNAs, was isolated from the human hepatocytes following treatment, using the miRNeasy kit (Qiagen, Valencia, CA) with optional on‐column DNase treatment included in the purification. Expression of 754 miRNAs was measured using the Taqman OpenArray Human miRNA Panel with an NT Cycler (Applied Biosystems, Foster City, CA). Each subject's RNA was analyzed on 2 different OpenArrays to yield technical duplicates. Threshold cycles were set manually based on visual inspection of the real‐time amplification curves of each individual miRNA. Final analysis of technical duplicates was completed within a single project to ensure that the same adjusted threshold was applied to each pair. C_T_ values were transformed to positive values (40‐C_T_) to ensure appropriate directionality of effect for the correlation analyses with RNA‐seq data. The remaining miRNA bioinformatics analyses mirrored that described in a previous analysis of this data set for evaluation of transport protein changes.^5^

### 2.3 Bioinformatic analysis of the RNA‐seq data

RNA‐seq library construction, EZBead preparation, and NextGen sequencing were performed using standard methods as described previously^6^ and used to measure UGT, SULT, NAT, TPMT, and GST mRNAs and compared to vehicle control (0.1% methanol). UGT1A genes were identified and quantified by unique exons 1 as exons 2‐5 are shared across this gene subfamily. The RNA‐Seq data analysis included quality assessment and sequence alignment prior to differential gene expression analysis as described previously.^6^ In brief, SOLiD Instrument Control Software and Experiment Tracking Software were used for read quality recalibration. Each sequence was scanned for low‐quality reads and any read length of less than 35 bases was discarded to effectively eliminate low‐quality reads while retaining high‐quality regions. BFAST was used as the primary sequence alignment algorithm employing a TopHat‐like strategy to align sequencing reads that crossed splicing junctions. Sequence reads were aligned to a filtering index to exclude sequences that were not of interest (eg, repeats and ribosomal RNA). Analyses were restricted to uniquely aligned sequences with 2 or less mismatches. Differentially expressed genes were identified using edgeR following exclusion of genes with less than 1 read per million mappable reads in more than half of samples. A generalized linear model considering the effects of individual donors as a random effect was used to identify gene expression levels directly affected by rifampin treatment. The *P*‐values were calculated for each gene and Benjaminin and Hochberg's algorithm was used to control the false discovery rate. Data reported in the primary tables and figures only for genes up‐ or downregulated >1.25‐fold by rifampin and *P *<* *.05. Clustering of mRNA expression changes and hepatocyte donors depicted in dendrograms were determined, using Euclidian distances and the complete linkage clustering method. Data visualization and hierarchical cluster analysis were performed with R software (build 3.2.3) and R Studio (v. 0.99.491), using the gplots and ggplots2 packages.

### 2.4 Rifampin treatment of human renal proximal tubule cells

Immortalized normal human proximal tubular kidney (NHPTK) cells^24^ were maintained in REGM media (Lonza, Basel, Switzerland) supplemented with 10% fetal bovine serum (Hyclone, GE Healthcare Bio‐Sciences, Pittsburgh, PA, USA) to maintain appropriate renal phenotype. NHPTK cells were maintained at 37°C in 95% humidified atmosphere (5% CO_2_). Studies were performed on cells in passages 6‐9 (corresponding to passage 3‐6 post‐immortalization) with individual passages considered a biological replicate (n = 4). NHPTK cells were treated with rifampin (10 μmol/L) or vehicle control (0.01% methanol) for 24 hours. Following treatment, ~1 million cells were washed in ice‐cold PBS, recovered via centrifugation, and the resultant pellet stored at −80°C pending RNA isolation.

### 2.5 Quantitative real‐time PCR of renal cells

Total RNA was extracted, using the miRNeasy Plus Mini Kit (Qiagen, Hilden, Germany) manufacturer protocol. UGT1A1, 1A6, 1A9, and 2B7, expression levels were determined via qRT‐PCR using GAPDH as an endogenous control. RNA quantification and quality were assessed, using optical spectrometry ratios (260/280 and 260/230 nm); mRNA was reverse transcribed to cDNA using the iScript Reverse Transcription Kit (Bio‐Rad, Hercules, CA) and diluted to obtain 25 ng/mL final cDNA concentration. Here, qRT‐PCR was performed on an Applied Biosystems Quantum Studio Viia 7 system with iTaq Universal SYBR Green (Bio‐Rad) and custom made primers (Life Technologies). The thermocycler parameters were 95°C for 30 seconds, then 40 cycles consisting of 95°C for 15 seconds followed by an annealing temperature for 30 seconds. Primer sequences and annealing temperatures are provided in Table S1. The delta–‐delta C_T_ method was applied to determine the relative expression of each gene for rifampin and vehicle‐treated cells as previously described.^5^ The fold change in gene expression is represented as the mean ± SEM of the biological replicates (n = 4).

### 2.6 ChIP‐seq PXR‐binding site in silico analysis

The conjugating enzyme genes in this study were evaluated in silico for PXR‐binding sites using a publically available ChIP‐Seq database generated using HepG2 cells treated with vehicle (dimethyl sulfoxide, DMSO) or rifampin.^25^ The in silico ChIP‐Seq testing was conducted as described previously.^5^ Promoter regions were specified as ±2 kb based upon the coordinates of each transcription start site.

### 2.7 Accession numbers

Raw RNA‐seq data were made publicly available through the National Center for Biotechnology Information Gene Expression Omnibus (GEO) database and can be accessed, using GEO series accession number GSE799933 (http://www.ncbi.nlm.nih.gov/geo/query/acc.cgi?acc=GSE79933). OpenArray miRNA data were made publically available through the Indiana University Center for Computational Biology and Bioinformatics and can be accessed at http://compbio.iupui.edu/group/6/pages/rifampin.

### 2.8 Physiologically based modeling and simulation

The potential clinical impact of rifampin induction of UGT1A4‐mediated midazolam N‐glucuronidation was evaluated via PBPK modeling and simulation, using the SimCYP population‐based simulator (version 15.1; SimCYP Limited, Sheffield, UK). The midazolam SimCYP library file was modified to describe the clinically observed disposition of the UGT1A4‐mediated N‐glucuronide metabolite of midazolam. The midazolam N‐glucuronide compound file was linked to the parent compound and designated as “Primary Metabolite 1” within the software. Midazolam N‐glucuronide model development was accomplished, using clinical data previously acquired during the control phase of a healthy volunteer (n = 12) herbal product–drug interaction study.^26^ SimCYP model parameters are available in Table S2. Simulated pharmacokinetic outcomes within 30% of observed endpoints were deemed sufficiently accurate to proceed with interaction simulations. Drug–drug interactions resulting from coadministration of rifampin (600 mg/day orally for 3 days) with midazolam (5 mg orally on day 3) were simulated in 10 virtual trials of 10 healthy volunteers (ages 20–50 years, 50:50 male:female). Initial simulations evaluated only the impact of a fivefold increase in the hepatic UGT1A4 mediated metabolism of midazolam achieved semimechanistically using the “UGT scalar” option within SimCYP. Subsequent simulations incorporated the impact of a simultaneous fivefold increase in UGT1A4 metabolism along with mechanistic description of rifampin‐induced changes in CYP3A4 activity. The multiple dose rifampicin library file within the SimCYP model was used as provided to describe rifampin induction of CYP3A. The only exception was that the maximal fold induction (Ind_max_) for CYP3A4/5 was set to 22.7, the mean value observed in the current hepatocyte experiments. The directly observed fold mRNA change was utilized based upon the assertion that appropriate use of higher Ind_max_ values improves model prediction accuracy of drug–drug interactions mediated via CYP3A4 induction.^27^

## 3 RESULTS

### 3.1 Rifampin regulation of hepatocyte drug metabolizing gene expression

**Figure 1:**
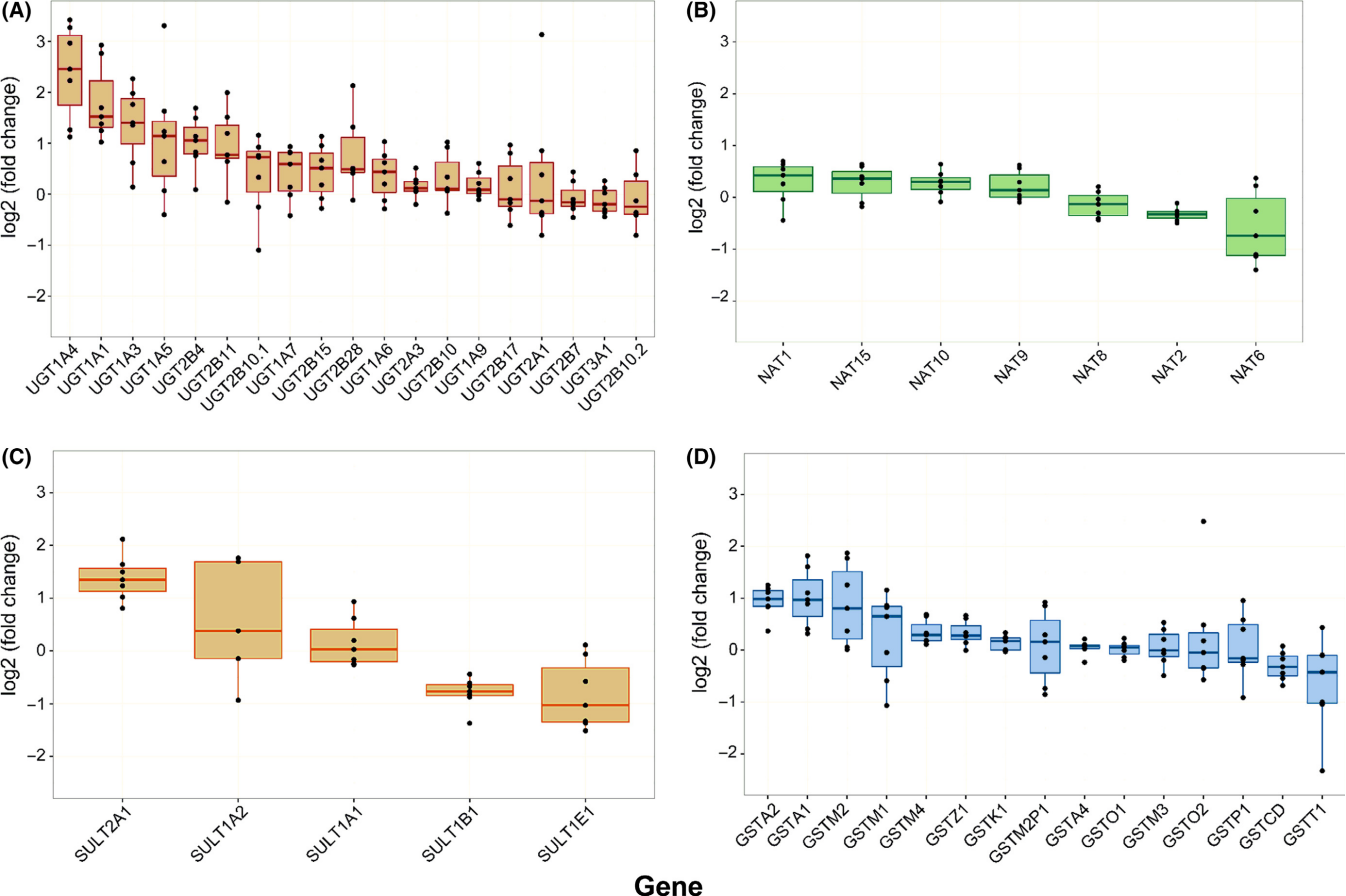
Individual changes in mRNA expression grouped by gene family; (A) UDP‐glucuronsyltransferases (UGTs), (B) N‐acetyltransferases (NATs), (C) sulfotransferases (SULTs), and (D) glutathione‐S‐transferases determined via RNA‐seq. Dots denote individual observed data points for each biological replicate (n = 7). Boxes denote observed median and interquartile range (IQR), whereas whiskers depict 1.5 times the IQR.

The effects of rifampin on the hepatocyte expression of 53 phase II drug metabolizing enzyme enzymes was evaluated by differential mRNA expression. The effects of rifampin on selected enzymes (greater than 1.25‐fold change in mRNA expression and *P *<* *.05) are outlined in Table 1. Rifampin treatment significantly induced the expression of 13 genes and repressed the expression 3 genes. UGT1A5 expression was induced by ~twofold in agreement with previous reports of rifampin induction (3.5‐fold)^28^ in human hepatocytes. UGT family mRNA expression was isoform dependent and induced or not changed in response to rifampin treatment (Figure 1). However, multiple members of the UGT1A enzyme family consistently demonstrated induction in response to rifampin treatment across all 7 hepatocyte donors (Figure S1). Here, mRNA expression of 3 NAT isoforms appeared to be repressed in response to rifampin treatment with the remaining isoforms largely unchanged (Figure 1). SULT2A1 mRNA expression was induced while SULT1B1 and 1E1 expression was repressed (Figure 1). SULT1E1 and SULT1B1 mRNA expression were repressed to a similar extent, a result consistent with reported coregulation of these 2 genes.^29^ Changes in GST mRNA expression were modest with mixed induction and repression observed (Figure 1). Consistent with previous report,^3^ TPMT mRNA expression was unchanged by rifampin treatment. Observed changes in mRNA expression were largely consistent across biological replicates with the exception of strong induction of UGT2A1 and GSTO2 observed only in hepatocytes from donor 5 (Figure S1). UGT1A4 and UGT1A1 were most strongly induced suggesting the possibility of clinically relevant drug–drug interactions resulting from concomitant rifampin administration with drug substrates of these enzymes and prompting further evaluation via physiologically based modeling and simulation.

**Table 1:** Effect of rifampin on the expression of selected conjugative drug metabolizing enzymes in human hepatocytes

Gene	Fold change^a^	*P*‐value	FDR	Examples of substrates
Upregulated				
UGT1A4	4.93	9.85 × 10^−113^	1.14 × 10^−109^	Amitriptyline, endoxifen, imipramine, midazolam
UGT1A1	3.19	2.82 × 10^−70^	1.64 × 10^−67^	Acetaminophen, bilirubin, SN‐38, raltegravir
SULT2A1	2.44	2.78 × 10^−44^	9.59 × 10^−42^	Androgens, dehydroepiandrosterone
UGT1A3	2.40	2.76 × 10^−30^	7.06 × 10^−28^	Ezetimibe, naproxen, quercetin
UGT1A5	2.07	8.79 × 10^−17^	1.15 × 10^−14^	1‐hydroxypyrene, 4‐methylumbelliferone, scopoletin
GSTA1	1.92	1.51 × 10^−21^	2.72 × 10^−19^	Busulfan, chlorambucil, thiotepa, androstene‐3,17‐dione
UGT2B4	1.89	3.54 × 10^−24^	7.30 × 10^−22^	Lorazepam, bile acids, carvedilol
GSTA2	1.87	3.80 × 10^−14^	3.57 × 10^−12^	Busulfan, dibenzopyrene diolepoxide
UGT2B11	1.76	1.27 × 10^−3^	1.32 × 10^−2^	12‐hydroxyeicosatetraenoic acid (HETE), 15‐HETE
GSTM2	1.69	6.63 × 10^−3^	4.71 × 10^−2^	1‐chloro‐2,4‐dinitrobenzene
GSTM1	1.68	1.65 × 10^−7^	6.08 × 10^−6^	Artemisinin
SULT1A2	1.61	2.16 × 10^−2^	1.07 × 10^−1^	Minoxidil, β‐napthol
UGT2B15	1.28	1.70 × 10^−4^	2.64 × 10^−3^	Acetaminophen, (S)‐oxazepam, tolcapone
Downregulated				
SULT1B1	−0.55	2.72 × 10^−18^	4.09 × 10^−16^	1‐napthol, 4‐nitrophenol, tri‐iodothyronine
SULT1E1	−0.57	9.87 × 10^−10^	5.30 × 10^−8^	Estrogen, naringenin, 4‐hydroxytamoxifen, curcumin
NAT2	−0.75	5.06 × 10^−5^	9.41 × 10^−4^	Dapsone, sulfasalazine, isoniazid

FDR, false discovery rate.

a Rifampin/control; reported only for genes up‐ or down‐regulated >1.25‐fold and *P *<* *0.05.

### 3.2 Conjugating enzymes and CYP450 isoforms appear to be coordinately regulated

Coordinate regulation of CYPs, UGTs, and transporters has been proposed as a defense mechanism providing protection against various chemical stressors.^30^ Correlation analysis suggests that several conjugating enzymes are coordinately regulated in response to rifampin treatment. As expected, multiple UGT genes displayed strong positive correlations in rifampin‐induced expression changes (Table 2). UGT1A4 and UGT2B15 expression changes positively correlated with changes in UGT2B4 expression. Our results are in agreement with the literature reported protein pairs of UGT1A4/2B4 (r_s_=.71, *P *<* *.0001, n = 82) and UGT2B4/2B15 (r_s_ = .63, *P *<* *.0001, n = 83) from a recent meta‐analysis.^31^ Interestingly, expression changes of multiple GST enzymes were negatively correlated with changes in UGT1A and 2B expression (Table 2). Changes in UGT1A1 expression correlated positively with changes in CYP3A7 and CYP2B6 expression while UGT1A5 expression negatively correlated with changes in CYP3A5 and CYP2E1 (Table 3). Interestingly, significant correlations were not observed between the major CYPs (eg, CYP1A2, 2C8, 2C9, and 3A4/5) and UGTs (UGT1A1, 1A3, 1A4, 1A9, 2B7). These data provide further evidence for coordinate regulation of drug metabolizing enzymes in response to rifampin treatment.

**Table 2:** Correlations of rifampin‐induced changes in the mRNA expression among the conjugating enzymes

Gene 1	Gene 2	Correlation coefficient^a^	*P*‐value
Positive correlation			
GSTA1	SULT2A1	.96	.003
UGT1A3	UGT2B4	.96	.003
GSTM2	GSTM4	.93	.007
SULT1B1	UGT1A5	.89	.012
UGT2B15	UGT2B4	.89	.012
SULT1E1	UGT2B17	.86	.024
SULT2A1	UGT2B15	.86	.024
UGT1A3	UGT2B15	.86	.024
UGT1A3	UGT2B17	.86	.024
GSTA1	UGT2B15	.82	.034
UGT1A3	UGT1A5	.82	.034
UGT1A4	UGT2B4	.82	.034
UGT1A5	UGT2B17	.82	.034
UGT1A9	UGT2B11	.82	.034
GSTA1	SULT1A2	.79	.048
NAT2	SULT1A1	.79	.048
Negative correlation			
GSTT1	UGT2B4	−.96	.003
GSTT1	UGT1A3	−.93	.007
GSTA4	UGT1A1	−.86	.024
GSTM1	UGT2B11	−.86	.024
GSTA1	GSTT1	−.82	.034
GSTO1	UGT1A4	−.82	.034
GSTT1	UGT2B15	−.82	.034
GSTA1	GSTM2	−.79	.048
GSTZ1	UGT1A4	−.79	.048

a Spearman correlation as assumption of normal distribution could not be made. Data recovered via RNA‐seq.

Only significant correlations (*P *<* *0.05 by Spearman correlation) are reported for those conjugating enzymes modulated by rifampin (>1.25 mean fold change, FDR <0.05).

**Table 3:** CYP450 genes correlated with clinically relevant conjugating enzyme genes

Gene 1	Gene 2	Correlation coefficient^a^	*P*‐value
Positive correlation			
UGT1A1	CYP3A7	.96	.003
UGT1A5	CYP1B1	.86	.024
UGT1A9	CYP1A1	.86	.024
UGT2B4	CYP2D6	.86	.024
SULT1B1	CYP1B1	.82	.034
NAT2	CYP2J2	.82	.034
UGT1A1	CYP2B6	.79	.048
Negative correlation			
GSTM1	CYP4F2	−.86	.024
UGT1A5	CYP3A5	−.79	.048
UGT1A5	CYP2E1	−.79	.048

a Spearman correlation as assumption of normal distribution could not be made. Data recovered via RNA‐seq. Only significant correlations reported (*P *<* *0.05 by Spearman correlation).

### 3.3 Changes in miRNA and conjugating enzyme mRNA expression are highly correlated

Correlation analyses between changes in miRNA expression and mRNA changes were performed to identify miRNAs that may regulate conjugating enzyme expression. Typically, miRNAs are expected to downregulate target gene expression which would result in a negative correlation in this analysis. However, changes in miRNA expression were both positively and negatively correlated with conjugating enzyme mRNA expression (Table 4), similar to a previous report of miRNA and CYP450 correlations.^6^ Five of the miRNA/RNA pairs identified, using correlation analysis were also predicted via TargetScan (Release 7.1).^32^ hsa‐miR‐200b was negatively correlated with SULT1A1, SULT1A2, and NAT2 (Table 4); consistent with downregulation of those genes by the miRNA. Rifampin‐induced expression of hsa‐miR‐200b may underlie the observed repression of NAT2 (Figure 1B, Table 1). The hsa‐miR‐766 was previously predicted in silico to target the HNF4α nuclear receptor^6^ which may explain the observed correlations with changes in UGT1A3, UGT2B4, UGT2B15, GSTO1, and GSTT1 mRNA expression. The vast miRNA and transcription factor network that controls the expression of the various conjugating enzymes likely underlies the observed positive and negative correlations.

**Table 4:** Conjugating enzyme‐miRNA pair correlations consistent with miRNA modulation of conjugating enzyme gene expression in response to rifampin treatment

miRNA	Gene 2	Correlation coefficient^a^	*P*‐value
Positive correlation			
hsa‐miR‐638	GSTT1	.99	.0004
hsa‐miR‐766	GSTT1	.96	.003
hsa‐miR‐92a	UGT1A9	.93	.007
hsa‐miR‐335	GSTCD	.93	.007
hsa‐miR‐342‐3p	GSTA4	.93	.007
hsa‐miR‐92a	UGT2B11	.89	.012
hsa‐miR‐92a	UGT2B7	.89	.012
hsa‐miR‐30d^c^	GSTM4^b^	.86	.024
hsa‐miR‐660	GSTA4	.86	.024
hsa‐miR‐320	UGT3A1^b^	.86	.024
hsa‐miR‐616	SULT1A1	.86	.024
hsa‐miR‐200a	GSTZ1	.86	.024
hsa‐miR‐200a	GSTO1	.86	.024
hsa‐miR‐21	GSTA4	.86	.024
hsa‐miR‐886‐3p	GSTM2P1	.82	.034
hsa‐miR‐92a	TPMT^b^	.82	.034
hsa‐miR‐320	SULT1A1	.82	.034
HSA‐MIR‐1180	NAT15	.82	.034
hsa‐miR‐361	NAT1	.79	.048
hsa‐miR‐92a	GSTCD	.79	.048
hsa‐miR‐30d^c^	GSTM2	.79	.048
hsa‐miR‐660	GSTT1	.79	.048
hsa‐miR‐21	GSTT1	.79	.048
Negative correlation			
hsa‐miR‐766	UGT1A3	−.96	.003
hsa‐miR‐148b^c^	GSTO1	−.96	.003
hsa‐miR‐200b^c^	SULT1A1	−.9	.006
hsa‐miR‐766	UGT2B4^b^	−.93	.007
hsa‐miR‐18a	UGT2B17	−.89	.012
hsa‐miR‐200b^c^	NAT2	−.85	.016
hsa‐miR‐107	NAT1	−.86	.024
hsa‐miR‐660	SULT1A2	−.82	.034
hsa‐miR‐638	GSTA1	−.82	.034
hsa‐miR‐638	UGT2B15	−.82	.034
hsa‐miR‐25	GSTZ1	−.82	.034
hsa‐miR‐18a	UGT2B15	−.82	.034
hsa‐miR‐23a^c^	UGT2B10^b^	−.82	.034
hsa‐miR‐744	UGT2A3	−.82	.034
hsa‐miR‐766	GSTA1	−.79	.048
hsa‐miR‐766	UGT2B15	−.79	.048
hsa‐miR‐218	GSTZ1	−.79	.048
hsa‐miR‐31	GSTO1	−.79	.048
hsa‐miR‐200b^c^	SULT1A2	−.76	.049

a Spearman correlation as assumption of normal distribution could not be made. Only significant correlations reported (*P *<* *.05 by Spearman correlation). Only those genes and miRNAs significantly altered by rifampin treatment (up‐ or down‐regulated >1.25‐fold and *P *<* *.05) were included in the correlation analyses.

b Denotes genes predicted via TargetScan to be targets of the correlated miRNA.

c Correspond to the nonpredominantly expressed miRNA sequence. TPMT, thiopurine S‐methyltransferase.

### 3.4 Rifampin modulation of UGT gene expression is cell line specific and appears to be largely PXR dependent

Rifampin treatment did not significantly alter the expression of UGT1A1, 1A6, 1A9, or 2B7 in NHPTK cells. The observed fold changes ranged from 0.95 to 1.04, consistent with a previously reported lack of PXR expression in this cell line.^5^ In Silico ChIP‐Seq analysis of rifampin‐treated HepG2 cells found PXR peaks within the promoter regions of 4 clinically relevant conjugating enzyme genes: UGT1A4, UGT1A6, SULT2A1, and GSTO1. ChiP‐Seq analysis was in agreement with the RNA‐seq results demonstrating increased gene expression of UGT1A4 and SULT2A1, suggesting a PXR‐mediated induction process.

### 3.5 Physiologically based modeling and simulation suggests that UGT induction contributes to observed rifampin–drug interactions with dual CYP3A/UGT substrates

**Figure 2:**
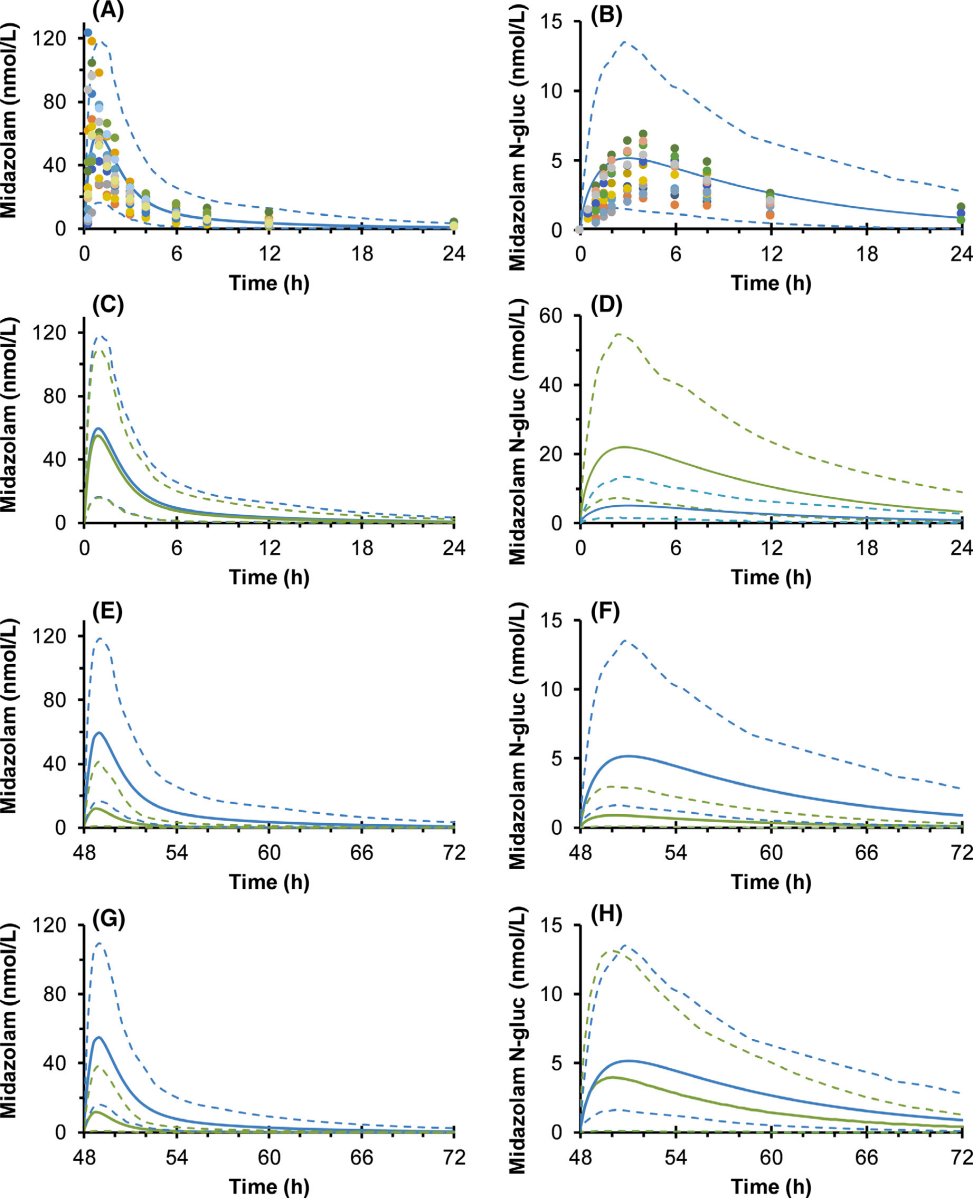
Physiologically based pharmacokinetic model predicted effects of rifampin‐induced midazolam metabolism. Observed and predicted midazolam (A) and midazolam N‐glucuronide (B) concentration‐time profiles following oral administration of midazolam (5 mg) to healthy volunteers (n = 12). Predicted midazolam and N‐glucuronide concentration‐time profiles considering the effects of rifampin coadministration (600 mg/day orally for 3 days) resulting in the following: UGT1A4 induction only (C and D), CYP3A induction only (E and F), and simultaneous induction of both UGT1A4 and CYP3A (G and H). Blue and green lines correspond to midazolam and N‐glucuronide disposition when taken alone or with rifampin, respectively. Solid and dashed lines denote the predicted arithmetic mean and 95% confidence intervals, respectively. Dots denote observed individual data; each color represents data from a single healthy volunteer (n = 12). PBPK, physiologically based pharmacokinetic

Simulated midazolam and midazolam N‐glucuronide concentration‐time profiles closely approximated clinically observed disposition and pharmacokinetic outcomes (Figure 2A and B, Table 5). Simulations evaluating the impact of rifampin‐induced UGT1A4 metabolism in isolation predicted markedly increased midazolam N‐glucuronide exposure (~fourfold) with minimal reductions in parent midazolam exposure (~10%), consistent with midazolam clearance‐mediated primarily by CYP3A4 (Figure 2C and D, Table 5). CYP3A4 induction only was predicted to reduce systemic midazolam exposure by nearly 10‐fold, in concordance with previous clinical and PBPK model‐predicted reports of hepatic CYP3A4 induction.^27^ Midazolam N‐glucuronide exposure was also predicted to be substantially reduced by rifampin, reflective of drastically reduced parent midazolam exposure leading to reduced substrate availability for UGT1A4‐mediated N‐glucuronidation. Simultaneous evaluation of UGT1A4 and CYP3A4 induction predicted >10‐fold mean reduction in plasma midazolam exposure but only ~2‐fold reduction in midazolam N‐glucuronide exposure, suggesting that the effects of limited substrate availability are partially overcome by simultaneous UGT1A4 induction.

**Table 5:** Model‐predicted rifampin mediated drug–drug interaction with midazolam

Midazolam		Midazolam N‐glucuronide	
Control (no interaction)			
AUC_obs_	209 (160‐274)	AUC_obs_	54.7 (45.3‐66.0)
AUC_pred_	185 (162‐213)	AUC_pred_	51.7 (44.9‐59.7)
$C_{\max_{\mathrm{obs}}}$	74.2 (56.5‐97.5)	$C_{\max_{\mathrm{obs}}}$	4.17 (3.46‐5.03)
$C_{\max_{\mathrm{pred}}}$	50.0 (44.1‐56.6)	$C_{\max_{\mathrm{pred}}}$	4.25 (3.73‐4.84)
Rifampin UGT induction only			
AUC_ind_	165 (144‐188)	AUC_ind_	219 (192‐250)
1/AUC_ratio_	1.12 (0.98‐1.28)	AUC_ratio_	4.24 (3.22‐5.57)
$C_{\max_{\mathrm{ind}}}$	46.7 (41.3‐52.8)	$C_{\max_{\mathrm{ind}}}$	18.5 (16.4‐20.9)
1/$C_{\max_{\mathrm{ratio}}}$	1.07 (0.95‐1.21)	$C_{\max_{\mathrm{ratio}}}$	4.35 (3.39‐5.62)
Rifampin CYP3A induction only			
AUC_ind_	19.1 (15.4‐23.7)	AUC_ind_	5.43 (4.36‐6.77)
1/AUC_ratio_	9.72 (8.48‐11.2)	1/AUC_ratio_	9.52 (8.30‐10.9)
$C_{\max_{\mathrm{ind}}}$	7.44 (6.01‐9.22)	$C_{\max_{\mathrm{ind}}}$	0.54 (0.43‐0.66)
1/$C_{\max_{\mathrm{ratio}}}$	6.71 (5.87‐7.67)	1/$C_{\max_{\mathrm{ratio}}}$	7.94 (9.08‐6.93)
Rifampin CYP3A and UGT induction			
AUC_ind_	18.4 (14.9‐22.8)	AUC_ind_	24.8 (20.0‐30.78)
1/AUC_ratio_	10.1 (9.32‐11.1)	1/AUC_ratio_	2.08 (2.24‐1.94)
$C_{\max_{\mathrm{ind}}}$	7.23 (5.85‐8.94)	$C_{\max_{\mathrm{ind}}}$	2.48 (2.02‐3.05)
1/$C_{\max_{\mathrm{ratio}}}$	6.91 (6.33‐7.69)	1/$C_{\max_{\mathrm{ratio}}}$	1.71 (1.59‐1.85)

Observed data recovered from a healthy volunteer (n = 12) study in which participants were administered a single oral dose (5 mg) of midazolam. AUC_obs_, observed area under the plasma concentration‐time curve (nmol/L × hours); AUC_pred_, predicted area under the plasma concentration‐time curve (nmol/L × hours); AUC_Ind_, predicted area under the plasma concentration‐time curve following rifampin induction (nmol/L × hours); AUC_ratio_, rifampin treatment:control ratio; $C_{\max_{\mathrm{obs}}}$, maximal observed plasma concentration (nmol/L); $C_{\max_{\mathrm{pred}}}$, maximal predicted concentration (nmol/L); $C_{\max_{\mathrm{ratio}}}$, rifampin treatment:control ratio; $C_{\max_{\mathrm{ind}}}$, predicted maximal concentration following rifampin induction (nmol/L). Values denote geometric mean and 95% confidence intervals.

## 4 DISCUSSION

Successful mitigation of CYP450 metabolic liabilities during drug development has increased the importance of non‐CYP450 enzyme contributions to drug metabolism. However, evaluation of non‐CYP450 mediated xenobiotic metabolism continues to pose research, development, and regulatory challenges.^33^ Standardized in vitro models to assess conjugating enzyme contributions to xenobiotic clearance and approaches to predict clinical consequences are evolving. As a result, mechanistic understanding of these metabolic pathways and reports of the effects of modulators of conjugating enzyme activity are relatively limited compared to the CYP450 system. This report outlines the effects of rifampin induction of conjugating enzyme expression and potential modulation via induced miRNA expression. These data: (1) show that several clinically relevant conjugating enzymes are inducible by rifampin; (2) support the association of rifampin‐induced miRNA modulation of conjugating enzyme expression; (3) indicate that UGT induction is cell‐line dependent; and (4) suggest the potential clinical relevance of UGT induction by rifampin.

Of the non‐CYP450 enzymes that contribute to xenobiotic metabolism, UGTs are the most important in terms of abundance in hepatic and extrahepatic tissues as well as in the wide range of xenobiotics including many drugs and endobiotics they metabolize. Drug–drug interactions mediated via UGT induction are inherently difficult to predict from in vitro data.^33^ LC‐MS/MS approaches to directly quantify UGT protein content within in vitro systems and human tissues^34^, ^35^, ^36^, ^37^, ^38^, ^39^, ^40^ have enhanced in vitro–‐in vivo extrapolation of UGT mediated metabolism. Future studies of UGT induction could leverage combinatorial approaches (eg, RNAseq and LC‐MS/MS) to provide complementary information. UGT1A1 has been previously demonstrated to be induced by treatment with dietary polyphenols including resveratrol, curcumin, and chrysin in Caco‐2 cells,^41^, ^42^ human hepatocytes,^43^ and PXR reporter assays.^44^ However, rapid metabolism and minimal systemic exposure of many dietary polyphenols may limit their ability to induce hepatic UGTs in vivo.^45^ In contrast, rifampin and other prototypic drug inducers are expected to produce systemic exposure sufficient to result in clinically relevant enzyme induction.^46^ Rifampin induction of UGT1A5 has been reported previously in human hepatocytes. Detection of hepatic UGT1A5 protein in human livers not exposed to rifampin is limited, suggesting that hepatic UGT1A5 is expressed only in response to xenobiotic exposure.

In agreement with previous report, SULT2A1 mRNA expression was induced while SULT1A1 was not altered by treatment with rifampin.^47^ Consistent repression of SULT1E1 and SULT1B1 gene expression was not expected to result from rifampin treatment particularly when considering previous reports of the interaction between rifampin and ethinyl estradiol.^18^ However, repression of SULT1E1 and SULT1B1 may result from the complex interplay of multiple rifampin‐induced transcription factors, similar to the mechanism described for rifampin repression of CYP7A1 expression.^48^ This may suggest that rifampin induces regulatory elements that act to suppress SULT1E1 and SULT1B1 mRNA expression, such as miRNAs or transcription repressors, or mechanisms other than rifampin activation of PXR may underlie the observed changes.

Expression of NAT2 mRNA was the most strongly repressed gene by treatment with rifampin. The mechanistic basis for this observation remains to be elucidated. Nevertheless, this novel in vitro observation may provide an alternate explanation for the clinically observed increase in hepatotoxicity that results from coadministration of rifampin with the NAT2 substrate isoniazid.^49^, ^50^ The mechanistic underpinnings of this drug–drug interaction have been the source of some debate as it does not appear to be related directly to induction of CYP450‐mediated reactive metabolite concentrations in humans or PXR‐humanized mice.^51^, ^52^ Mouse models suggest that human PXR modulates hepatotoxicity associated with rifampin and isoniazid via increased accumulation of an endogenous hepatotoxin.^51^ However, human NAT2 genetic polymorphisms that result in a slow acetylator phenotype have been strongly associated with increased risk of isoniazid hepatotoxicity. It then leads that perhaps rifampin down‐regulation of NAT2 is creating a drug‐induced slow acetylator phenotype that leads to increased risk of isoniazid hepatotoxicity when administered with rifampin. Rifampin‐induced formation of hydrazine from isoniazid has been posited to underlie increased hydrazine plasma levels observed in patients taking rifampin and isoniazid as compared to those taking isoniazid alone.^53^ Alternatively, repressed NAT2 activity leading to impaired hydrazine elimination, or a combination of both increased formation and reduced elimination, may explain the apparent increase in hydrazine exposure caused by rifampin. Further reduction in limited NAT2 activity by rifampin could potentially explain reports of increased incidence of hepatotoxicity when slow acetylators take isoniazid and rifampin.^54^

The alpha‐class GSTs catalyze the GSH‐dependent detoxification of several alkylating chemotherapy agents and numerous environmental pollutants.^55^ GST induction has also been suggested, using high‐sensitivity real‐time PCR^3^ and likely represents another defense mechanism against xenobiotic exposure. The observed changes in GST expression measured via RNAseq are in alignment with previous reports using alternate quantification approaches.

Several miRNAs have been suggested to modulate conjugating enzyme expression and function. miR‐376c was identified as a modulator of UGT2B15 and UGT2B17 in prostate cancer cell lines.^10^ This observation was later confirmed and expanded to include miR‐331‐5p, miR‐376c‐3p (formerly miR‐368‐1) for UGT2B15 and miR‐376c, miR‐409, and miR‐494 for UGT2B17.^9^ miR‐216b has been identified in silico as a potential modulator of several UGT2B family members, including UGT2B15.^7^ Similarly, miR‐491‐3p is associated with UGT1A1 expression and activity changes in hepatic cell lines^8^ However, the expected inverse correlation between the levels of miR‐491‐3p and UGT1A1 mRNA were not demonstrated in a panel of 38 normal livers. More recently, a functional genomics approach assessed the complete compliment of miRNAs that could regulate UGT1A expression and identified 6 additional miRNAs (miR‐21‐3p, miR‐200a‐3p, miR‐103b, miR‐1286, miR‐376b‐3p, and miR‐141‐3p) that decrease UGT1A‐dependent activity.^13^ SULT1A1 expression in human liver has been associated with miR‐631 expression levels.^56^ miR‐133a has been associated with repression of GSTP1 mRNA and protein in lung and bladder cancer cell lines^57^, ^58^ while miR‐133b has been associated with repressed GSTP1 mRNA expression in prostate cancer cell lines.^59^ miR‐513a‐3p has also been associated with repressed GSTP1 expression in lung cancer cells.^60^ An inverse correlation between PXR translational efficiency and miR‐148a has also been reported.^61^ Interestingly, none of these miRNAs were revealed by our correlation analysis. This may be the result of both direct and indirect mechanisms mediated via rifampin induction. However, the miRNA‐mRNA pairs identified in Table 4, particularly those predicted via TargetScan, may represent a reasonable starting point to better understand the interplay of miRNA modulation and transcription factor activation via functional and mechanistic studies.

Induction of UGT1A4 observed in vitro prompted evaluation via PBPK modeling and simulation. An available dataset which included the UGT1A4 mediated N‐glucuronide metabolite of midazolam was utilized to evaluate the potential contribution of isolated and simultaneous CYP450 and UGT1A4 induction. It is acknowledged that induction of midazolam N‐glucuronidation is unlikely to be of clinical significance owing to the relatively minor contribution of UGT1A4 to overall midazolam clearance. However, this exemplar drug–drug interaction highlights the potential importance of considering simultaneous induction of alternate pathways, particularly for drugs where the fraction metabolized by CYP450 is relatively lower than that of midazolam. Including induction of all known metabolic pathways into predictive PBPK drug–drug interaction models may also help alleviate the systematic tendency to under predict the magnitude of drug–drug interactions resulting from induction.

These data reveal differential effects of rifampin on the human conjugating enzyme transcriptome and potential associations with miRNAs. The magnitude of phase 2 enzyme mRNA induction in response to rifampin was relatively lower than that observed for induction of CYP450 enzymes. This may be the result of a relatively lesser contribution of PXR‐mediated induction to the overall induction potential of the conjugating enzymes evaluated. We acknowledge that mRNA expression changes may not directly reflect changes in protein content and activity. Further studies are needed to evaluate the correlations between rifampin‐induced mRNA expression changes, miRNA modulation, and enzyme activity as posttranscriptional and posttranslation modifications may alter this relationship. However, this global expression approach was aimed at revealing additional factors that might contribute to regulation of important drug metabolizing enzymes. These findings should inform future studies to elucidate and quantitatively predict the impact of epigenetic regulation and conjugating enzyme induction on clinical drug disposition.

## AUTHOR CONTRIBUTIONS

Participated in research design: Gufford, Liu, Desta, and Skaar; while Gufford and Eadon conducted experiments. Contributed new reagents or analytical tools: Eadon; performed data analysis: Gufford, Robarge, Lin, Gao, Liu, and Skaar. Lastly, Gufford, Eadon, Robarge, Liu, Desta, and Skaar wrote or contributed to writing of the manuscript.

## DISCLOSURES

None declared.

## Supporting information


**Figure S1.** Hierarchical clustering of rifampin‐induced changes in conjugating enzyme mRNA expression determined via RNA‐seq. Clustering of mRNA expression changes and hepatocyte donors depicted by the dendrograms were determined, using Euclidian distances and the complete linkage clustering method. Red = induced genes; blue = repressed genes.
**Table S1.** Primer sequences and annealing temperatures
**Table S2.** SimCYP model input parameters for midazolam and midazolam N‐glucuronide
