# Supplementary material for: Rifampin modulation of xeno‐ and endobiotic conjugating enzyme mRNA expression and associated microRNAs in human hepatocytes
Source: Pharmacol Res Perspect. 2018 Mar 26;6(2):e00386. doi: 10.1002/prp2.386 (PMC5869567; doi:10.1002/prp2.386)
Supplement: Supplementary file 1 — Figure S1. Hierarchical clustering of rifampin‐induced changes in conjugating enzyme mRNA expression determined via RNA‐seq. Clustering of mRNA expression changes and hepatocyte donors depicted by the dendrograms were determined, using Euclidian distances and the complete linkage clustering method. Red = induced genes; blue = repressed genes. Table S1. Primer sequences and annealing temperatures Table S2. SimCYP model input parameters for midazolam and midazolam N‐glucuronide [file PRP2-6-e00386-s001.docx]

**Rifampin Modulation of Xeno- and Endo-Biotic Conjugating Enzyme mRNA Expression and Associated MicroRNAs in Human Hepatocytes**

**Supplemental Data**

Brandon T. Gufford, Jason D. Robarge, Michael T. Eadon, Hongyu Gao, Hai Lin, Yunlong Liu, Zeruesenay Desta, and Todd C. Skaar

Department of Medicine, Division of Clinical Pharmacology (B.T.G., J.D.R, M.T.E, Z.D., T.C.S.) and Department of Medical and Molecular Genetics (H.G., H.L., Y.L.), Indiana University School of Medicine, Indianapolis, Indiana

**Supplemental Figure 1.**  Hierarchical clustering of rifampin-induced changes in conjugating enzyme mRNA expression determined via RNA-seq. Clustering of mRNA expression changes and hepatocyte donors depicted by the dendrograms were determined using Euclidian distances and the complete linkage clustering method. Red = induced genes; blue = repressed genes.

**
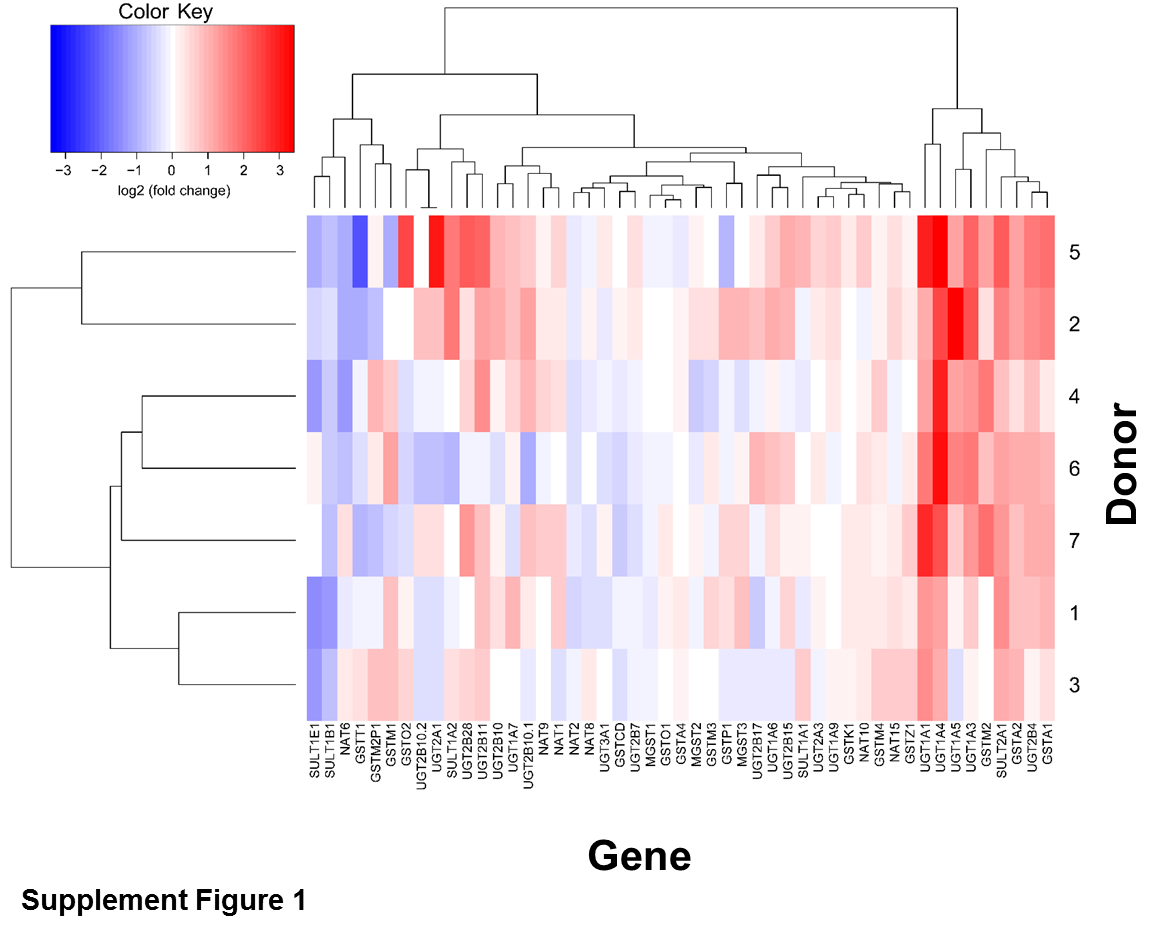
**

**Supplemental Table 1: Primer sequences and annealing temperatures**

| Gene | Forward Primer | Reverse Primer | Temp. (^o^C) |
| --- | --- | --- | --- |
| *GAPDH* | TGCACCACCAACTGCTTAGC | GGCATGGACTGTGGTCATGAG | 60 |
| *UGT1A1* | CCTTGCCTCAGAATTCCTTC | ATTGATCCCAAAGAGAAAACCAC | 57 |
| *UGT1A6* | CAACTGTAAGAAGAGGAAAGAC | ATTGATCCCAAAGAGAAAACCAC | 51 |
| *UGT1A9* | GAACATTTATTATGCCACCG | ATTGATCCCAAAGAGAAAACCAC | 54 |
| *UGT2B7* | GGAAATCATGTCAATATTTGG | CATTGTCTCAAATAATGTAGTG | 48 |

**Supplemental Table 2: SimCYP Model Input Parameters for Midazolam and Midazolam N-glucuronide**

| **Midazolam** | | **Rifampicin** | | **Midazolam N-glucuronide** | |
| --- | --- | --- | --- | --- | --- |
| Route | Oral | Route | Oral | **PhysChem and Blood Binding** | |
| Dose Units | Dose (mg) | Dose Units | Dose (mg) |  |  |
| Dose | 5.000 | Dose | 600.000 | Mol Weight (g/mol) | 501.890 |
| Start Day | 3.000 | Start Day | 1.000 | log P | 2.500 |
| Start Time | 9h0m | Start Time | 9h0m | Compound Type | Ampholyte |
| Dosing Regimen | Single Dose | Dosing Regimen | Multiple Dose | pKa 1 | 6.200 |
|  |  | Dose Interval (h) | 24.000 | pKa 2 | 3.500 |
| **PhysChem and Blood Binding** | | Number of Doses | 3.000 | BP input type | User |
|  |  |  |  | B/P | 1.000 |
| Mol Weight (g/mol) | 325.800 | **PhysChem and Blood Binding** | | Haematocrit | 45.000 |
| log P | 3.530 |  |  | fu Input | User |
| Compound Type | Ampholyte | Mol Weight (g/mol) | 823.000 | fu | 0.032 |
| pKa 1 | 10.950 | log P | 4.010 | Reference Binding Component | HSA |
| pKa 2 | 6.200 | Compound Type | Ampholyte | Protein Reference Conc (g/L) | 45.000 |
| BP input type | User | pKa 1 | 1.700 | % Bound to Lipoprotein | 0.000 |
| B/P | 0.603 | pKa 2 | 7.900 | % Bound to Lipoprotein (CV %) | 0.000 |
| Haematocrit | 45.000 | BP input type | User |  |  |
| fu Input | User | B/P | 0.900 | **Absorption** | |
| fu | 0.032 | Haematocrit | 45.000 |  |  |
| Reference Binding Component | HSA | fu Input | User | fu(Gut) | 1.000 |
| Protein Reference Conc (g/L) | 45.000 | fu | 0.116 |  |  |
| % Bound to Lipoprotein | 0.000 | Reference Binding Component | HSA | **Distribution** | |
| % Bound to Lipoprotein (CV %) | 0.000 | Protein Reference Conc (g/L) | 45.000 |  |  |
|  |  | % Bound to Lipoprotein | 0.000 | Distribution Model | Full PBPK Model |
| **Absorption** | | % Bound to Lipoprotein (CV %) | 0.000 | Replacement Organ? | No |
|  |  |  |  | Organ Replaced | n/a |
| Absorption Model | 1st order | **Absorption** | | User-defined Additional Organ | No |
| Input type | User |  |  | Type | n/a |
| fa | 1.000 | Absorption Model | 1st order | Vss input type | Predicted |
| CV fa (%) | 23.300 | Input type | Predicted | Prediction Method | Method 1 |
| ka (1/h) | 1.420 | lag time (h) | 0.000 | Concentration-dependent volume | No |
| CV ka (%) | 19.000 | CV lag time (%) | 30.000 | log Po:w | 2.500 |
| lag time (h) | 0.000 | fu(Gut) input type | User | fut input type | Predicted |
| CV lag time (%) | 30.000 | fu(Gut) | 1.000 | logDvo:w (pH=7.4) input type | Predicted |
| fu(Gut) input type | User | Q(Gut) Input | User | logP vo:w input type | Predicted |
| fu(Gut) | 1.000 | Q(Gut) (L/h) | 9.420 | Compound Type | Ampholyte |
| Q(Gut) Input | Predicted | CV Q(Gut) (%) | 30.000 | pKa 1 | 6.200 |
| Peff,man Type | n/a | Peff,man Type | Global | pKa 2 | 3.500 |
| Permeability Assay | PCaco-2 | Peff,man (10-4 cm/s) | 2.151 | B/P | 1.000 |
| Apical pH : Basolateral pH | 7.4 : 7.4 | Permeability Assay | PCaco-2 | Haematocrit | 45.000 |
| Activity | Passive & Active | Apical pH : Basolateral pH | 6.5 : 7.4 | fu | 0.032 |
| PCaco-2(10E-06 cm/s) | 213.000 | Activity | Passive | Adipose input type | Predicted |
| Reference Compound | Multiple | PCaco-2(10E-06 cm/s) | 15.000 | Bone input type | Predicted |
| Reference Compound Value (10E-06 cm/s) | 0.000 | Reference Compound | Propranolol | Brain input type | Predicted |
| Scalar | 0.290 | Reference Compound Value (10E-06 cm/s) | 21.150 | Gut input type | Predicted |
|  |  | Scalar | 1.000 | Heart input type | Predicted |
| **Distribution** | |  |  | Kidney input type | Predicted |
|  |  | **Distribution** | | Liver input type | Predicted |
| Distribution Model | Minimal PBPK Model |  |  | Lung input type | Predicted |
| SAC kin (1/h) | 0.200 | Distribution Model | Minimal PBPK Model | Muscle input type | Predicted |
| SAC kout (1/h) | 0.250 | SAC kin (1/h) | 0.000 | Skin input type | Predicted |
| SAC CLin (L/h) | 10.162 | SAC kout (1/h) | 0.000 | Spleen input type | Predicted |
| SAC CLout (L/h) | 4.641 | SAC CLin (L/h) | 0.00 | Pancreas input type | Predicted |
| Volume [Vsac] (L/kg) | 0.23 | SAC CLout (L/h) | 0.00 | Kp Scalar | 1.000 |
| Vss input type | User | Volume [Vsac] (L/kg) | 0.00 |  |  |
| Vss (L/kg) | 0.880 | Vss input type | User | **Elimination** | |
| CV Vss (%) | 30.000 | Vss (L/kg) | 0.420 |  |  |
| Liver input type | User | CV Vss (%) | 50.000 | Allometric Scaling | Not Used |
| Liver Kp | 1.000 | Liver input type | User |  |  |
|  |  | Liver Kp | 1.000 | Clearance Type | In Vivo Clearance |
| **Elimination** | |  |  | CL (po) (L/h) | 4.500 |
|  |  | **Elimination** | | CV CL (po) (%) | 30.000 |
| Allometric Scaling | Not Used |  |  | Active Uptake into Hepatocyte | 1.000 |
|  |  | Allometric Scaling | Not Used | CL R (L/h) | 0.300 |
| Clearance Type | Enzyme Kinetics |  |  |  |  |
| FI Correction | Not used | Clearance Type | In Vivo Clearance |  |  |
| PLR Correction | Not Used | CL (iv) (L/h) | 8.700 |  |  |
| In vitro metabolic system | Recombinant | CL (iv) CV | 30.000 |  |  |
|  |  | Active Uptake into Hepatocyte | 1.000 |  |  |
| Pathway | 1-OH | CL R (L/h) | 1.260 |  |  |
| Enzyme | CYP3A4 |  |  |  |  |
| Vmax | 5.230 | CYPs and/or UGTs Interaction |  |  |  |
| Km | 2.160 |  |  |  |  |
| fu mic | 1.000 | Enzyme | CYP2C8 |  |  |
|  |  | Ki (µM) | 24.500 |  |  |
| Pathway | 1-OH | fu mic | 1.000 |  |  |
| Enzyme | CYP3A5 |  |  |  |  |
| Vmax | 19.700 | Enzyme | CYP3A4 |  |  |
| Km | 4.160 | Ki (µM) | 15.000 |  |  |
| fu mic | 1.000 | fu mic | 1.000 |  |  |
|  |  | Ind max | 22.700 |  |  |
| Pathway | 4-OH | CV (%) | 30.000 |  |  |
| Enzyme | CYP3A4 | MIA (pmol/mg microsomal protein) | 5700.447 |  |  |
| Vmax | 5.200 | Ind C50 (µM) | 0.320 |  |  |
| Km | 31.800 | CV (%) | 30.000 |  |  |
| fu mic | 1.000 | fu inc | 1.000 |  |  |
|  |  | γ | 1.000 |  |  |
| Pathway | 4-OH |  |  |  |  |
| Enzyme | CYP3A5 | Enzyme | CYP3A5 |  |  |
| Vmax | 4.030 | Ind max | 22.700 |  |  |
| Km | 34.800 | CV (%) | 30.000 |  |  |
| fu mic | 1.000 | MIA (pmol/mg microsomal protein) | 5015.225 |  |  |
|  |  | Ind C50 (µM) | 0.320 |  |  |
| In vitro metabolic system | Recombinant | CV (%) | 30.000 |  |  |
|  |  | fu inc | 1.000 |  |  |
| Pathway 1 | Pathway 1 | γ | 1.000 |  |  |
| Enzyme | UGT1A4 |  |  |  |  |
| Vmax | 445.000 |  |  |  |  |
| Km | 40.300 |  |  |  |  |
| fu mic | 1.000 |  |  |  |  |
| rUGTSystem | User |  |  |  |  |
| rUGTScalar - Liver | 5.000 |  |  |  |  |
| rUGTScalar - Intestine | 0.000 |  |  |  |  |
| rUGTScalar - Kidney | 1.000 |  |  |  |  |
| Forms Metabolite | Pri Met 1 |  |  |  |  |
|  |  |  |  |  |  |
| Feed formed renal metabolite into kidney | No |  |  |  |  |
|  |  |  |  |  |  |
| Use Allelic variants for Enzyme 1 | No |  |  |  |  |
| Enzyme | CYP2C9 |  |  |  |  |
|  |  |  |  |  |  |
| Use Allelic variants for Enzyme 2 | No |  |  |  |  |
| Enzyme | CYP1A2 |  |  |  |  |
|  |  |  |  |  |  |
| Ontogeny Profile | No Profile Used |  |  |  |  |
| Biliary CLint (Hep) (µL/min/10^6^) | 0.000 |  |  |  |  |
| CV Biliary CLint (Hep) (%) | 30.000 |  |  |  |  |
| Ontogeny Profile | No Profile Used |  |  |  |  |
| Active Uptake into Hepatocyte | 1.000 |  |  |  |  |
| CL R (L/h) | 0.085 |  |  |  |  |
